# Reduced decision bias and more rational decision making following ventromedial prefrontal cortex damage

**DOI:** 10.1016/j.cortex.2021.01.015

**Published:** 2021-05

**Authors:** Sanjay Manohar, Patricia Lockwood, Daniel Drew, Sean James Fallon, Trevor T-J Chong, Deva Sanjeeva Jeyaretna, Ian Baker, Masud Husain

**Affiliations:** a Nuffield Dept of Clinical Neurosciences, University of Oxford, UK; b Centre for Human Brain Health, University of Birmingham, UK; c Dept of Experimental Psychology, University of Oxford, UK; d National Institute for Health Research Bristol Biomedical Research Centre, University Hospitals, Bristol NHS Foundation Trust and University of Bristol, UK; e Turner Institute for Brain and Mental Health, Monash University, Victoria 3800, Australia; f Department of Neurology, John Radcliffe Hospital, Oxford, UK; g Department of Neurosurgery, John Radcliffe Hospital, Oxford, UK

## Abstract

Human decisions are susceptible to biases, but establishing causal roles of brain areas has proved to be difficult. Here we studied decision biases in 17 people with unilateral medial prefrontal cortex damage and a rare patient with bilateral ventromedial prefrontal cortex (vmPFC) lesions. Participants learned to choose which of two options was most likely to win, and then bet money on the outcome. Thus, good performance required not only selecting the best option, but also the amount to bet. Healthy people were biased by their previous bet, as well as by the unchosen option's value. Unilateral medial prefrontal lesions reduced these biases, leading to more rational decisions. Bilateral vmPFC lesions resulted in more strategic betting, again with less bias from the previous trial, paradoxically improving performance overall. Together, the results suggest that vmPFC normally imposes contextual biases, which in healthy people may actually be suboptimal in some situations.

## 1 Introduction

Decision biases are a central part of human cognition. They make us behave in ways that might be considered irrational (Akerlof & Dickens, 1982; Tversky & Kahneman, 1974). Indeed, some biases actively involve irrationally incorporating information into decisions. For example, people tend to repeat an action they have recently performed (Braun et al., 2018), indicating that information about previous decisions is maintained and integrated into future actions, even when this might be inappropriate. There is evidence that such biases are underpinned by specific brain processes (De Martino et al., 2006; Wimmer & Shohamy, 2012), raising the possibility that damage to the brain could, paradoxically, mitigate biases (Akrami et al., 2018; Kapur, 1996) and lead to more rational decision making.

Ventromedial prefrontal cortex (vmPFC) – broadly defined as including medial orbitofrontal cortex (OFC), subgenual cingulate and the posterior part of frontopolar cortex on the medial surface–is strongly implicated in subjective preferences, valuation, confidence, and moral decision-making in humans (Bechara et al., 2000; Chan et al., 2016; O'Doherty, 2011; Rolls, 2015). Patients with vmPFC lesions may have changes in personality and social cognition, yet show variable deficits on standard value-based choices (Clark et al., 2008; Eslinger & Damasio, 1985; Levens et al., 2014; Pelletier & Fellows, 2019; Schneider & Koenigs, 2017; Vaidya & Fellows, 2015). One possibility is that computations in vmPFC contribute contextual nuances to decisions. For example, they might normally drive biases in decision making observed in healthy individuals. If this were the case, then lesions to this region might paradoxically make decision making more rational under some circumstances. Indeed a previous study demonstrated that some brain lesions can reduce the negative impact of previous outcomes on an investment decision (Shiv et al., 2005).

Here we investigated whether vmPFC might contribute to biases in human value-based decision making. A large body of functional neuroimaging work has demonstrated that vmPFC represents a range of value information from recent history and context (Hampton & O'Doherty, 2007). It is therefore possible that loss of these contextual value cues might *reduce* decision biases that are normally observed in humans when they make value-based decisions, and thereby lead to more rational decision making. To examine this we used a new version of a probabilistic reversal learning task. Many behavioural theories explain learning in choice tasks on the basis of participants estimating the values of available stimuli or actions. Reversal learning tasks require individuals to select the more rewarding of two stimuli, when the reward contingencies for the two stimuli may vary.

**Fig. 1:**
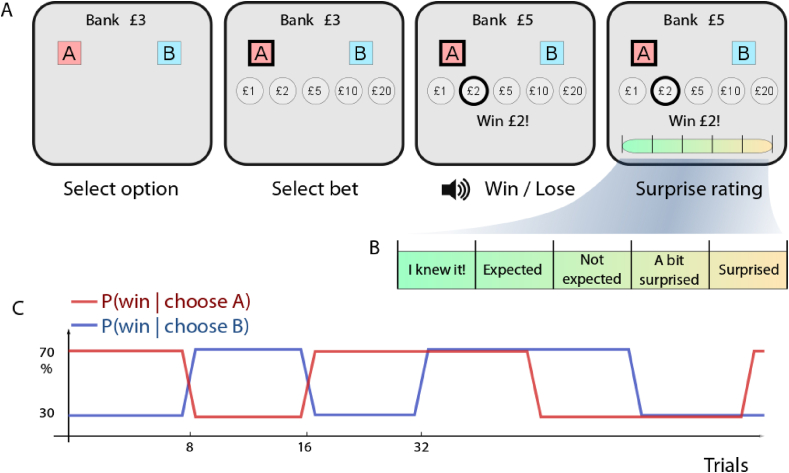
Reversal learning and Gambling Task. **A** Participants were required to select one of two options by touching one of the coloured squares. After making their decision, they had to decide how much money they would like to bet on this option. Subsequently they either won or lost the amount, and one of two sounds was played. **B** Afterwards, they were asked to indicate how surprised they were at the outcome, on each trial. This was rated on a scale of 5 points, ranging from “I knew it!” to “Surprised”. **C** Probability of winning after selecting a particular colour was either 30% or 70%, and this varied orthogonally for the two colours, with an option's value changing (reversing) on average every 12 trials.

In our paradigm, confidence in decision making was probed by asking participants to bet an amount of money on the choice they had made after each selection. Post-decision wagers have previously been used to study confidence monitoring in humans and animals on other tasks (Fleming et al., 2010; Hampton, 2001). In such scenarios, participants can take a gamble on their choice. We used a probabilistic learning task in which participants had to choose one of two options, and would either win or lose, so that they had to learn which option was better. After selecting an option, they made a post-decision wager, which determined how much was at stake, which we used as a metacognitive index (Fig. 1A). However, such bet-based measures could conflate decision confidence with expectation of reward (Schurger & Sher, 2008), so in order to separate these two out, values of the two options were varied orthogonally (Fig. 1C). Participants were explicitly instructed that sometimes one, both, or neither of the options would be likely to win, and thus they needed to bet wisely to win the most money. In this design, therefore, a rational agent's bets should reflect not just confidence in whether they chose the *better* option, but also whether the chosen option is *likely to win*. We can dissociate these by asking whether bets track the estimated value of the chosen option, rather than its value relative to the alternative.

To further quantify expectation of reward, after each outcome was revealed, participants were asked to rate their subjective surprise at winning or losing, on that trial (Fig. 1B). Bayesian surprise is a statistic reflecting how unexpected an outcome is relative to prior expectation. Subjective ratings of surprise may therefore allow us to quantify a person's insight into their own betting strategy. Reinforcement learning models were adapted to precisely quantify betting strategy and subjective surprise.

## 2 Results

Sixteen patients with unilateral mPFC lesions, one extremely rare case with bilateral vmPFC lesions (patient MJ) and 33 age-matched healthy controls were assessed.

**Fig. 2:**
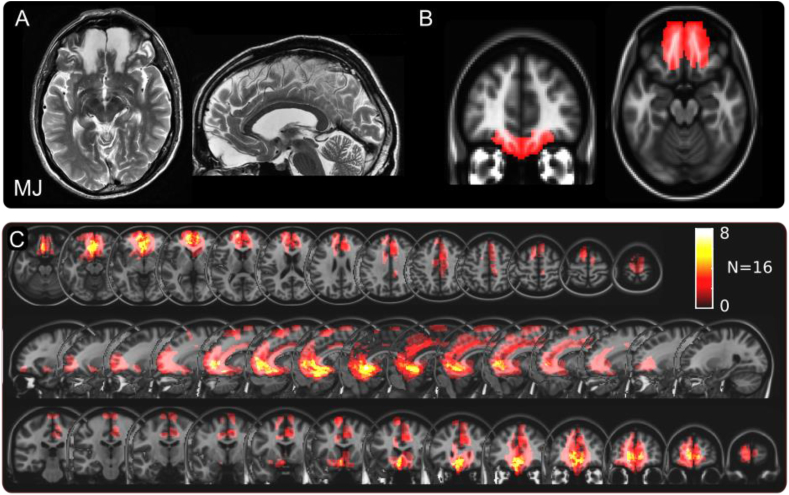
Medial prefrontal cortical (PFC) lesions. **A** Representative slices from T2-weighted MRI scan showing patient MJ's lesions, showing bilateral damage involving vmPFC/medial OFC. **B** MJ's lesions mapped onto MNI template. **C** Overlap map of lesions for the 16 patients with unilateral medial PFC damage.

MJ's lesions involved both medial OFC and frontopolar cortex (Fig. 2A). Notably the rest of the brain was spared on structural MRI. Comparison with the McGill-McConnell histological template (Mackey & Petrides, 2014) indicated his lesions primarily affected Brodmann Areas 11 and 14 bilaterally (left hemisphere: centroid MNI coordinates [-12, +46, −18] mm, right: [+13, +46,-18] mm, Table S1). The sixteen patients with isolated focal unilateral mPFC damage had previously suffered haemorrhages from an anterior communicating artery aneurysm and had a range of lesions involving the medial frontal lobe (Fig. 2C, left hemisphere: centroid [+4,+15,+7], right [-6,+20,+3]).

MJ was a 59-year-old right-handed man who developed personality disturbances after a traumatic brain haemorrhage sustained many years previously. Prior to this, he had obtained high grades in secondary education, a professional qualification and worked in a relatively demanding job. He presented to the clinic at the prompting of his new partner. Twenty-nine years previously he had been assaulted from behind and fell on railings, sustaining a frontal head injury with lobar haemorrhage. He was in a coma for two weeks, before gradually improving over the next 3 months. He returned to work, with minimal noticeable cognitive deficits, although acquaintances considered him to be inappropriately overfamiliar at times, for example hugging people he didn't know. He continued to hold his job without difficulty.

MJ remains behaviourally slightly disinhibited. In conversation, he may be flamboyant and socially very engaging. However, at other times he may become highly fixated on subjects, repeating topics or themes, and encountering difficulty in taking turns when speaking. He appears to have reduced empathy. At home, he can sometimes become angry for very little reason, having a temper outburst lasting up to an hour, and then subsequently wondering what all the fuss was about when his partner explains how upsetting it was. He encounters significant difficulty weighing up options when making a decision, especially when there are many options to choose from. For example at a restaurant, he may take more than half an hour to choose from a menu. His mood and levels of motivation are normal.

### 2.1 Supranormal performance after bilateral lesions

**Fig. 3:**
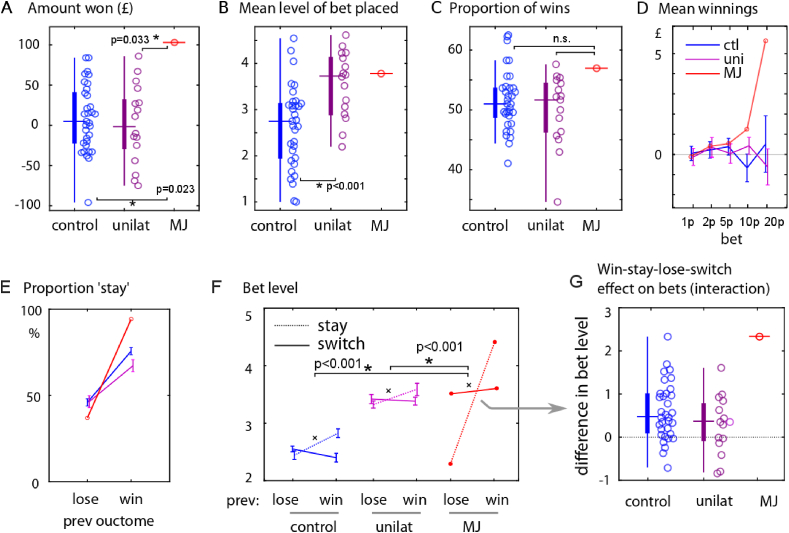
Behavioural results. **A** MJ earned more money on this task than any of the 33 control participants and 16 unilateral mPFC lesion patients. Horizontal tick indicates the median of each group, with box indicating the interquartile range. **B** Overall patients with mPFC lesions bet more on average than controls. Patient MJ was at 80th percentile on bet amount, in keeping with other mPFC patients. Overall bet amount therefore could not account for MJ's advantage on this task. **C** Accuracy on selecting the option most likely to win was no different between patients and controls, and was not different in MJ. Therefore better choices could not account for his advantage. **D** The amount won was split up according to the amount bet on that trial. MJ showed the greatest winnings when he bet high, in contrast to healthy controls or unilateral mPFC patients. This suggests his advantage was due to strategic betting. **E** Trials were split according to whether participants previously won or lost, and the proportion of trials on which the response was the same (“stay”) or different (“switch”) was calculated. There was no significant difference in win-stay-lose-switch strategy between the patients and controls. **F** The amount bet on different trials was split up according to whether the participant stuck to or switched their choice, and according to whether they won or lost on the previous trial. The mean level of bet in each of the four conditions is shown (with SEM). Healthy controls bet more after a win, but only when sticking to the same choice; when they switched they bet less (win-stay interaction, signified by × ). mPFC patients bet more overall, but otherwise showed the same strategy as controls. MJ showed a much stronger effect of previous wins on bet level, when he repeated the same option (stronger interaction term). **G** The interaction term from panel **F** for each subject is shown, such that a positive value indicates betting more on win-then-stay or lose-then-switch trials, compared to win-switch and lose-stay trials. MJ had a larger interaction term than any other participant.

First, we quantified simple performance measures for the groups. Remarkably, patient MJ performed *supranormally* on the task, outperforming everyone else tested. He won a total of £114, the highest amount won by any participant, including 33 controls and 16 unilateral vmPFC patients (Fig. 3A). Controls won on average £13.97 ± s.d. 40.6, and medial frontal patients £8.24 ± 50.0. MJ won significantly more than both groups (Z-test MJ vs. controls: Z = 2.49, p = .013; Z-test MJ vs. unilateral patients: Z = 2.11, p = .035).

This high score could have been driven either by a higher proportion of wins, or by strategically betting higher after choices more likely to win*.* Although mPFC patients, overall, bet more than healthy individuals (mean bet level 3.55 ± s.d. .70 vs 2.63 ± .87, unpaired t (47) = 3.70, p < .001), further analysis showed that MJ did not bet significantly more than unilateral mPFC cases (mean 3.76, Z = .31; Fig. 3B), so this alone cannot explain his higher winnings. MJ's proportion of wins was also no greater than that of controls (Figs. 3C and 57.0% vs 52.0% ± 5.1%, Z = 1.04, p = .23), suggesting that his betting pattern, rather than choice selection, was the critical factor.

To investigate why MJ won significantly more—despite winning no more often–we binned his winnings according to the amount bet on each trial (Fig. 3D). While most participants showed relatively flat curves, indicating no greater winnings when they bet higher, MJ had far more wins *when he bet high*, suggesting that strategic betting was responsible for his very high winnings. To further characterise this strategy, we examined betting after a win or loss, and according to whether participants chose the same (stay) or different (switch) option than they did on the previous trial. As expected people tended to repeat a choice more after a win than a loss (Fig. 3E) (arcsine-transformed proportion of stay trials after win vs loss, t (50) = 7.55, p < .001); but this win-lose difference did not differ significantly between unilateral mPFC patients and controls (two-sample *t* (48) = .93, p = .36), nor between MJ vs. controls (Z = 1.18, p = .24) or vs. unilateral patients (Z = 1.23, p = .22).

A common-sense strategy on this task might be to bet more after a win, especially when choosing the same option as before. Accordingly, the amount bet was modulated by the win-stay lose-switch interaction, with participants betting more when repeating a choice after a win (Fig.3F; 2 × 2 ANOVA previous win by stay, 2-way interaction of previous win x stay F (1,196) = 20.1, p < .001). They also bet more after a win (F (1,196) = 5.49, p = .020), but not significantly more when staying than switching (F (1,196) = 3.50, p = .063). Comparing the interaction term across groups (Fig. 3G), there was no significant difference in the win-stay betting effect in unilateral patients than controls (unpaired t (47) = 1.28, p = .21), but the bilateral patient strategically *bet more on win-stay-lose-switch* than other trials, compared to controls (interaction term Z = 3.05, p = .002, the largest of all participants) or unilateral mPFC patients (Z = 3.65, p < .001). Thus MJ's betting was considerably more strategic than other people.

### 2.2 Computational model of betting reveals healthy biases

To understand better the underlying pattern of participants' betting we examined value learning by fitting the standard Rescorla-Wagner learning rule to each individual's choices. This accurately predicted decisions on 77% of trials (s.d. 10%; range 56–99% across individuals). However, there were no differences in learning rate or decision noise, either between MJ vs unilateral mPFC patients, MJ vs controls or unilateral mPFC cases vs controls (Supplementary Fig.S1). This suggests that the observed higher betting rates in unilateral mPFC cases and MJ cannot simply accounted for by differences in value learning.

Further analysis comparing bets to trial-by-trial model estimates of value (Rutledge et al., 2014) revealed that healthy participants’ bets not only tracked the value of the chosen option, but were also *biased by their previous bets*, as well as the value of the *unchosen option*.

**Fig. 4:**
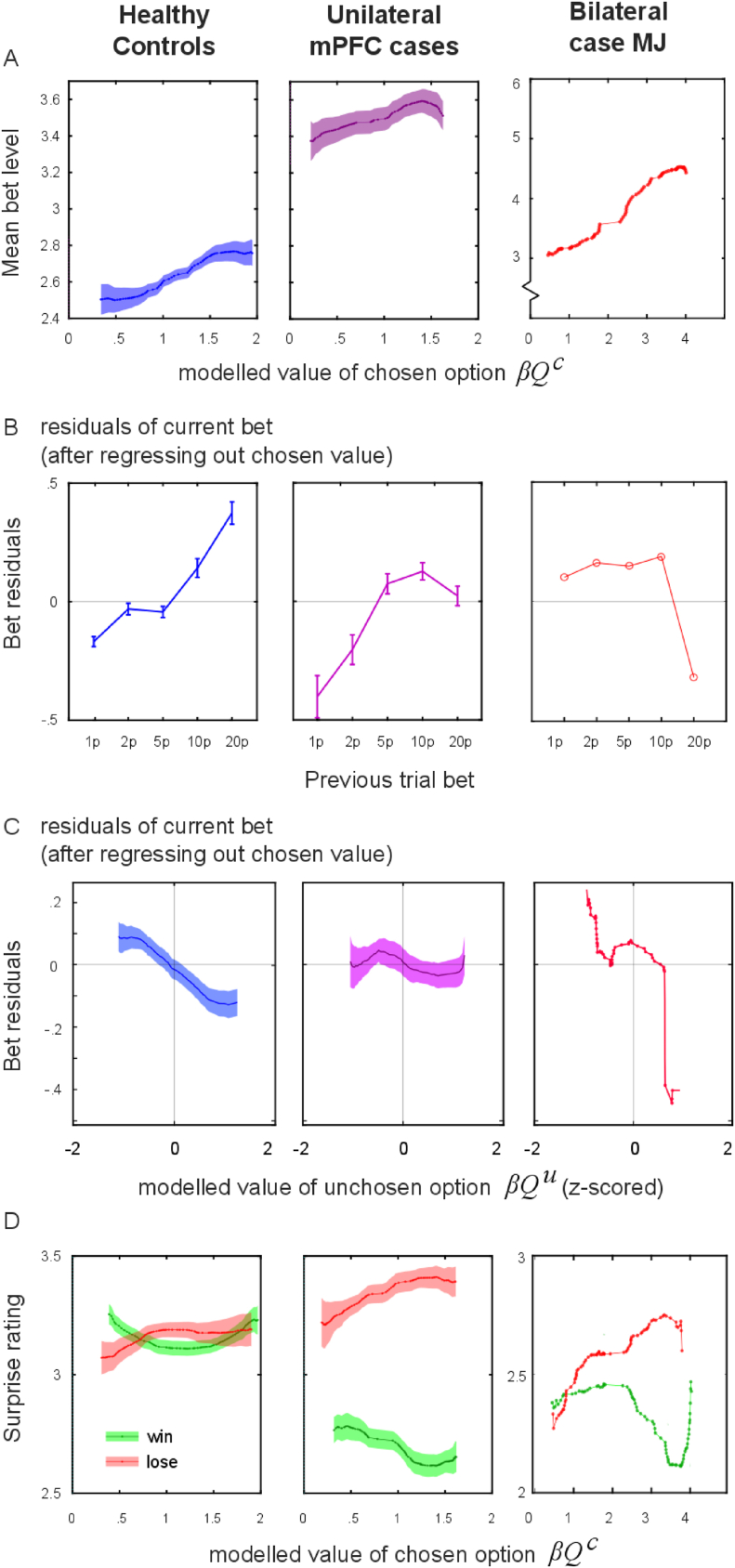
Betting is predicted by the modelled value and by previous bets. A Mean bet increased with modelled chosen value for all participants. Overall patients bet more than controls. Patient MJ had greater sensitivity (slope), increasing his bet with increased learnt value of the choice he made. (Bet levels assigned values 1 to 5, mean for each bin in a sliding window of width 25 percentiles over the range of modelled values, in 1% quantile steps; shading is standard error across subjects). B Bets were affected by the amount bet on the previous trial. To illustrate this visually, the effect shown in A was factored out using linear regression, and residuals shown, split by the level of bet on the previous trial. There was an interaction between group and effect of previous trial, with controls showing greater effects of previous bets. MJ had a significantly smaller effect of previous bets. C Bets were affected by the unchosen item's modelled value, after factoring out the chosen item's value. Healthy controls bet less when the other item was more valuable, i.e., they bet more when they were more confident in their decision. Unilateral mPFC lesion patients did not show this bias but MJ did. D Participants were more surprised if they lost after a higher-value choice, and when they won after a lower-value choice, as expected if surprise tracks absolute prediction error. This effect was present in both unilateral mPFC patients and MJ, but unilateral cases were less surprised at winning in general.

In general, participants bet higher when their chosen option had a higher learned value (Fig. 4A). In other words, their confidence in how likely they were to win increased systematically as the model's estimated value of the option they chose increased over trials. This relationship was tested using a linear mixed model, $bet_{t}\;∼\;1+\;Q_{t}^{c}+\left(1\;|\;subject\right)$, where$\;bet_{t}$ is the bet on trial *t*, on a scale of 1 to 5, and $Q_{t}^{c}$ is the modelled value of the chosen option on a given trial, based on the learning model (predictors z-scored within participants). Bets increased by .312 ± .018 per unit increase in choice value (t(4561) = 17.11, p < .001), but note that unilateral mPFC patients bet more in absolute terms (Fig. 4A).

Over and above this chosen value effect, betting was strongly dependent on bets on the *previous trial* (Fig. 4B; $bet_{t}\;∼\;1+\;Q_{t}^{c}+bet_{t-1}+\left(1\;|\;subject\right)$, effect of previous bet t (7140) = 19.1, p < .001). Adding the previous-bet term improved goodness of fit (change in Bayesian Information Criterion, ΔBIC = −332). To test whether this previous-trial bias was specific to the item chosen on the previous trial, the following model was tested: $bet_{t}\;∼\;1+\;Q_{t}^{c}+bet_{t-1}+stay+stay\times bet_{t-1}+\left(1|subject\right)$, where *stay* is an indicator (±1) for trials where the same response was chosen as the previous trial. There was no interaction of previous bet with staying on the same option (t (4837) = 1.59, p = .11), indicating that bets were biased by the previous trial irrespective of whether the same option was chosen, and adding this term worsened fit (ΔBIC = +1.15).

Although the modelled value of the chosen item is the optimal determinant of bet size, people's bets might also be affected by the value of the *unchosen* item. In other words, rather than rationally betting according to the chance of winning, their bet might be affected by decision confidence. In this case, bets would reflect how close the learned value of the unchosen item is to that of the chosen one (i.e. chosen minus unchosen value, $Q^{c}-Q^{u}$). Recall that in this version of probabilistic reversal learning, to perform well, participants must track the values of both the chosen and unchosen option independently. We fitted the model: $bet_{t}\;∼\;1+\;Q_{t}^{c}+Q_{t}^{u}+\left(1|subject\right)$, where $Q_{t}^{u}$ indicates the value of the unchosen option, z-scored within subjects. Bets decreased by .089 ± .013 per unit increase in unchosen value (Fig. 4C, t (4984) = 6.76, p < .001). This indicates that participants bet significantly more when they were more confident about their decision compared to the learned value of the *alternative option*, irrespective of the expected probability of winning.

### 2.3 Patients with lesions showed reduced biases

In comparison to controls, Patient MJ was less biased by previous bets. To test this we fitted the betting model across healthy controls and MJ: $bet_{t}\;∼\;\left(1+Q_{t}^{c}+bet_{t-1}\right)*patient+\left(1|subject\right)$ including the factor *patient* as an indicator for MJ in the same mixed model, and the ∗ operator indicates that the 2-way interactions are included. He showed smaller biases from the previous bet ($bet_{t-1}\times patient$ interaction, effect = 8.4% of the previous bet, t (4986) = 5.25, p < .001). Crucially, however, his data demonstrated larger effects of value on betting (Fig. 4A, value × patient interaction, .53 bet levels per unit value, t (4986) = 6.62, p < .001). To test whether the effect of decision confidence–taking into account both *chosen and unchosen* value–was different in MJ, the model: $bet_{t}\;∼\;\left(1+Q_{t}^{c}+Q_{t}^{u}\right)*patient+\left(1|subject\right)$ was used. MJ did not differ from controls in using the *unchosen* option's value when betting (unchosen value × patient interaction, t (6987) = .050, p = .96). Thus, overall his bets were more rational, driven more by the *expected chance of winning*, but not by previous bets.

We used a similar approach to examine betting in the unilateral mPFC lesion group. We fitted the same model to controls and unilateral patients together, where *patient* was now a group indicator for the unilateral mPFC lesion patients. Overall, unilateral mPFC patients bet significantly more than controls (main effect of group, t (6936) = 3.66, p = .001). They were less influenced by previous trial bets than controls (Fig. 4B, group × previous bet interaction, t (6936) = 4.63, p < .001). Unilateral mPFC cases were no more sensitive to *chosen* value than controls (no significant group × value interaction, t (6936) = 1.94, p = .053). Finally, unilateral patients were significantly less affected by the unchosen option than controls (Fig. 4C, $Q_{t}^{u}\times group$ interaction, t (6936) = 3.72, p < .001; controls −.094 bet units per unit change in value, compared to +.001 in the unilateral lesion group). Thus both the previous-bet and unchosen-item biases were weaker in these patients.

Subjective surprise ratings on a given trial increased as a function of the absolute reward prediction error (Fig. 4D). This was tested using the model $surprise_{t}\;∼\;1+R_{t}*Q_{t}^{c}+\left(1|subject\right)$, where the $R_{t}$ term indicates that surprise differed for win vs loss trials, and a negative $R_{t}\times Q_{t}^{c}$ interaction would indicate that wins are surprising after choosing a low-valued option, and vice versa for losses. As expected, there was a significant interaction, indicating that subjective surprise tracked the modelled absolute prediction error (interaction of reward × chosen value, t (6989) = 2.65, p = .008) with losses being more surprising than wins (main effect of reward, t (6989) = 8.17, p < .001) and no main effect of chosen value (t = .02, p = .98).

To compare subjective surprise ratings across groups, we modelled $surprise_{t}\;∼\;\left(1+R_{t}*Q_{t}^{c}\right)*patient+\left(1|subject\right)$. Comparison of MJ with controls revealed no significant difference in the absolute prediction error effect (reward × chosen value × group interaction: t (4984) = .91, p = .36) nor in the reward effect (reward × group interaction t (4984) = 1.67, p = .094). There were no other significant differences. For the unilateral mPFC group vs. controls, there was no difference in the absolute prediction error effect (3-way interaction, t (6985) = 1.30, p = .19), but unilateral mPFC patients were much less surprised by wins than losses, compared to controls (reward × group, t (6985) = 14.2, p < .001). There was no overall difference in surprise (main effect of group, t (6985) = .983, p = .33), and no group × chosen value interaction (t (6985) = .347, p = .729). Thus, unilateral patients were less surprised when they won, but there were no other group differences in reporting subjective surprise.

As in Fig. 4B, in this task we observed increased betting after wins, rather than loss chasing (Campbell-Meiklejohn et al., 2008), which was greatest after a low bet (Supplementary materials).

Finally, to address whether working memory or cognitive control factors might explain MJ's superior performance on the reversal learning task, in addition to formal neuropsychology, we used experimental behavioural tasks. MJ's performance did not differ significantly from controls on either a prosaccade or antisaccade task, indicating no significant deficits in processing speed or cognitive control (Table S2, data from controls and unilateral patients previously described in Manohar & Husain, 2016). Visuospatial working memory span (Supplementary materials) also showed no differences from controls.

## 3 Discussion

In this study we used a novel reversal learning task in which participants made post-decision wagers on their choices, thereby providing a measure of their confidence in winning, and also rated their surprise at outcomes (Fig. 1). Analysis was performed on both performance data as well as with a computational model of value learning. In healthy volunteers, bets tracked the expected chance of winning (Fig. 4A), but also showed strong biases: People's bets tended to be similar to their bets on the previous trial, and were higher when the *unchosen* option was less likely to win. Patients with unilateral mPFC lesions bet more overall (Fig. 3B), but showed weaker biases from the previous trial and from the unchosen option. The bilateral patient MJ also showed a weaker bias from the previous trial (Fig. 4B), but crucially had a stronger effect of the chosen option's probability of winning (Fig. 4A). This meant that he won more than any other healthy volunteer or unilateral patient on this task (Fig. 2A), despite no difference in learning which option was better. Thus, his performance can be seen as exhibiting a more rational betting strategy than in healthy people.

A large body of evidence has revealed that many aspects of human decision making are seemingly irrational, driven by biases that appear to lead to suboptimal outcomes (De Martino et al., 2006; Talluri et al., 2018; Tversky & Kahneman, 1974; Urai et al., 2019). Evidence that some of these biases are driven by normal cognitive operations underpinned by specific brain processes (De Martino et al., 2006; Wimmer & Shohamy, 2012) raises the possibility that damage to the brain might paradoxically reduce such biases (Akrami et al., 2018; Kapur, 1996) and perhaps lead to more rational behaviour. However, to date only limited causal evidence for such a possibility exists in humans (Greene, 2007; Knoch et al., 2006; Koenigs et al., 2007).

The findings presented here show that it is indeed possible for more rational decision making to emerge–at least on a value based reversal learning task–after bilateral vmPFC lesions. This is not to say that all decisions and behaviours become more rational after such brain damage. Clearly, although he managed to continue to work in a demanding job, patient MJ showed evidence of dysfunction in social cognition and some aspects of decision making and judgment in everyday life, just as previous reported cases (Bechara et al., 2000; Berlin et al., 2004; Eslinger & Damasio, 1985; Shamay-Tsoory et al., 2005).

There is some previous circumstantial evidence that mPFC lesions may reduce decision biases. For example, patients with mPFC damage show smaller biases in probabilistic estimation (O’Callaghan et al., 2018), reduced affective contributions to reasoning (Shamay-Tsoory et al., 2005), and may indeed make more utilitarian moral judgements, suggesting more rational valuation with less affective bias (Ciaramelli et al., 2007; Koenigs et al., 2007; Krajbich et al., 2009). These effects might be underpinned by a more general increase in rationality after damage to this region. One possible explanation for this is that individuals with vmPFC lesions might be free of *affective biases* that normally contribute to such decision making but this remains to be established.

In line with this, Shiv et al. (2005) asked patients with a variety of lesions (amygdala, orbitofrontal and insula) to opt in or out of gambles with positive expected value. Controls tended to opt out especially after a loss, whereas the patients continued to bet, thus winning more. This can be compared to our win-stay analysis (Fig. 3F), where MJ bet more than controls on win-stay choices, but did not bet less on lose-switch choices. Further evidence that biases can depend on specific brain areas comes from patients with insula damage, who may lose the normal tendency towards the gamblers’ fallacy (Clark et al., 2014). With this bias, participants tend to re-choose an option that previously lost (because the history of wins should balance out on average). Transcranial stimulation to lateral prefrontal cortex increases this bias (Xue et al., 2012). In our study, there is a possible analogy with the unchosen option effect (Fig. 4C), where people bet less when the alternative was valuable (perhaps also because the two options should balance out on average). Unilateral ventromedial patients lost this bias. However, in our task, lesions did not affect the option decisions themselves.

Biases from previous trials may rely on information retained in working memory. Thus an important null result is that the bilateral patient was unimpaired in working memory accuracy (Table S2). He had considerable difficulty remembering verbal lists (Table 1). This memory deficit might have contributed to his lack of trial history biases. However, against this possibility, performance was normal on a specific working memory task, suggesting that the previous bet effect was not simply memory-related. Furthermore, his normal learning and decision-making indicate he was integrating and retaining the specific value information involved in the biases, making memory deficits less likely to contribute. One interpretation of the loss of bias could be that medial frontal areas are required for normal integration of the biasing or interfering information into the current decision. An alternative interpretation is that normal biases are driven by suboptimal heuristics, and that medial frontal lesions abolish these heuristics.

**Table 1:**
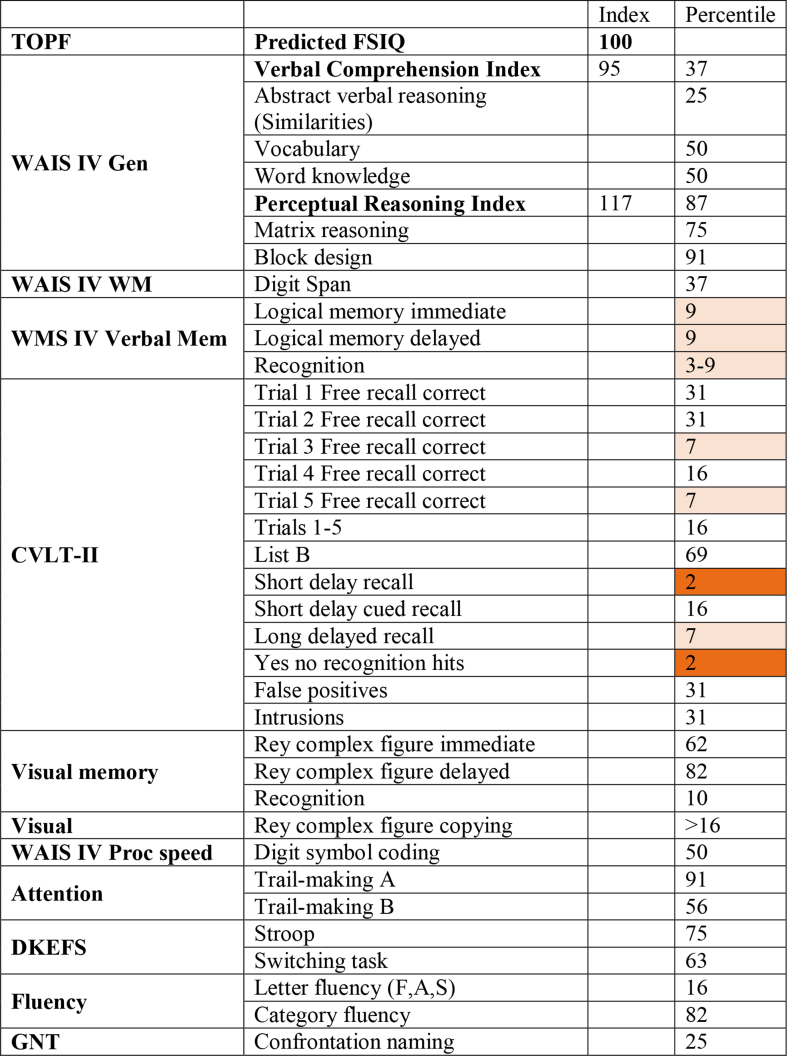
**Summary of standardised neuropsychological scores for patient MJ.** Impairments were seen in the verbal learning task for short delay recall and yes-no recognition. WAIS: Wechsler adult intelligence scale; WMS: Wechsler memory scale; WM: working memory; CVLT: California verbal learning test; DKEFS: Delis-Kaplan executive function system; GNT: Warrington graded naming test. Red indicates scores in the “extremely low” range (<2nd centile), and pink indicates scores in the borderline range (<10th centile).

Patients with vmPFC/OFC lesions have previously been shown to bet more under uncertainty (Clark et al., 2008), being generally less risk averse (Bechara et al., 2000; Levens et al., 2014), and our results directly support this finding. However, bets reflect a combination of general risk seeking, confidence, biases and strategic factors. In our study, increased betting alone was insufficient to explain the bilateral patient's advantage in this task. Instead, reduced biases may have permitted strategic betting, such as the hot hand effect or loss chasing. Interestingly, a previous study had identified that dorsomedial prefrontal lesions can *increase* the bias caused by eye movements during decisions (Vaidya & Fellows, 2015), but to our knowledge, no human studies have shown reduced biases after lesions in the way demonstrated here.

Information about unchosen options and recent actions may be disrupted by medial or orbitofrontal lesions (Buckley et al., 2009; Levens et al., 2014), which might thus account for the reduced biases in unilateral patients. The effect parallels a recent rodent study where parietal inactivation also paradoxically improved performance, by reducing the active bias from previous trials (Akrami et al., 2018). However, it is unclear why bilateral lesions did not attenuate this bias in MJ. Our finding of larger surprise differences between winning and losing may also match previous reports of increased emotional responses to stochastic outcomes after vmPFC lesions (Levens et al., 2014) and could parallel increases in reward sensitivity observed in these patients (Manohar & Husain, 2016).

Intriguingly, we found no consistent effects on option-selection in this task. Previous studies of classical reversal learning in patients with vmPFC lesions have shown varied effects. Patients tend to perseverate, maladaptively repeating their previous choices even after reward contingencies reverse (Fellows & Farah, 2003; Rolls et al., 1994), but other studies have found only a marginal effect (Daum et al., 1991), and yet others showed normal performance after unilateral lesions, but impaired reversal after bilateral lesions (Hornak et al., 2004). This is consistent with detailed studies in animals suggesting that impairments after OFC lesions may be mild, with medial lesions only impairing performance when discrimination is harder (Izquierdo et al., 2017; Rudebeck & Murray, 2011), and impairments potentially improved by further lesions (Stalnaker et al., 2007). Yet other work has demonstrated that vmPFC lesions produce unstable choices while preserving subjective valuation of single objects (Henri-Bhargava et al., 2012). However, we did not find any deficits in value-based selection of options in our task. This could be because the paradigm used here crucially tests the use of learned values, rather than subjective valuation or rule-following.

In non-human primate studies, brain areas encoding the values of options also encode decision confidence, such as OFC (Kepecs et al., 2008). In humans, fMRI activation increases with decision confidence in vmPFC (De Martino et al., 2013; Lebreton et al., 2015; Rolls et al., 2010; Yokoyama et al., 2010). Although some studies have demonstrated inaccurate confidence judgements after prefrontal lesions (Fleming et al., 2014), others find no deficits even with bilateral lesions (Lemaitre et al., 2018). Remarkably, disrupting anterior PFC with TMS can actually *improve* metacognitive confidence judgements (Shekhar & Rahnev, 2018). Thus, if medial PFC encodes variables that might bias valuation, lesions to this area should paradoxically improve performance in some situations, as observed here.

Of course, human lesion studies are inherently limited by the possibility of damage not visible on the MRI scans. Although all patients reported here had brain haemorrhages affecting the mPFC, with very little damage outside this region, the bilateral patient had suffered a traumatic injury, followed by haemorrhage. It is possible that this resulted in a different pattern of microscopic damage: although traumatic injuries may appear focal, often the functional damage can be quite widespread. This limits the conclusions that can be drawn about the causal role of medial frontal cortex specifically. However we suggest that the most likely explanation is the bilateral nature of his lesions: reward value is usually considered to be represented bilaterally in OFC (Hampton & O'Doherty, 2007; Rolls, 2015), suggesting that unilateral lesions are less likely to show manifest impairments. One difficulty with interpreting lesion studies is whether the changes reflect direct lesion effects, or compensatory strategies. The chronic nature of his lesion may be a key difference between MJ and other studies demonstrating deficits in reversal learning after vmPFC lesions (Fellows & Farah, 2003; Rolls et al., 1994). This may have allowed recovery and adaptation, leading to his strategic betting pattern. In this case, it is unclear whether it is vmPFC loss per se, or the network-level consequences of this, that attenuates biases. Functional imaging studies in patients might potentially shed light on this in the future.

In summary, the results suggest that vmPFC may drive biases in healthy people. A patient with bilateral lesions won more than other participants did, coupled with more strategic betting and reduced biases, which were attenuated in unilateral patients too. vmPFC may bring contextual information to influence action, which may be suboptimal in some situations.

## 4 Methods

### 4.1 Participants

All behavioural data, individual patients’ imaging lesion masks, and scripts to run the task are available at osf.io/4kfqz. We report how we determined our sample size in the unilateral group, all inclusion criteria, all manipulations and all measures in the study. There were no data exclusion criteria and no data exclusions.

We tested one patient with bilateral medial frontal lesions (Case MJ), along with 16 patients with unilateral mPFC lesions and 33 age-matched healthy controls. The 16 cases (9 female) with unilateral mPFC damage were selected from a database of 450 patients with anterior communicating artery aneurysms. These patients were a subset of those previously reported on a different task (Manohar & Husain, 2016), as only a proportion of those patients returned for follow up. Their mean age was 49.7 ± 10.2 years. 33 healthy age-matched volunteers were recruited from an advert. The mean age was 51.3 ± 18.5 years. The mean lesion volume was 17.5 ± 11.5 cm^3^, and lesion volume did not correlate with overall winning (Spearman ρ = .23, p = .39) or betting (ρ = .29, p = .27) across the group. Of the 33 healthy controls, 7 did not complete all 160 trials in the learning task, with a mean of 156.7 ± s.d. 8.3 trials completed. Of the 16 unilateral patients, 9 did not complete all trials, with a mean of 136.5 ± 31.2 trials completed. The bilateral patient completed all 160 trials.

### 4.2 Neuropsychological assessment of case MJ

Overall, neuropsychological assessment demonstrated MJ to have generally well-preserved intellectual abilities. He obtained a mildly reduced score on tests of verbal skills primarily reduced by difficulties with abstract verbal reasoning. With the exception of mildly reduced letter fluency performance (which might be attributable to previous dyslexia), performance on other tasks of attention and executive function were generally normal. Observationally, there is evidence of disinhibition, reduced empathy, egocentricity, and reduced insight.

In more detail: MJ's estimated premorbid level of functioning was in the average range based on lifetime reading abilities (TOPF predicted FSIQ = 100). Assessment of current general intellectual function produced a Verbal Comprehension Index (95) falling in the lower half of the average range. On a test of abstract verbal reasoning (Similarities) his score fell at the 25th centile and on the test of word knowledge at the 50th centile. His Perceptual Reasoning Index (117) fell in the high average range. With respect to memory, he could repeat five digits forwards and four in reverse (Digit Span = 37th centile). He struggled with both immediate and delayed recall of two narrative passages from the Wechsler Memory Scale (WMS-V), both scores falling at 9th centile. On the California verbal learning test II, little learning over trials was demonstrated, and he scored at 16th centile for total number of words recalled, with long delayed recall score falling at the 7th centile. He made a good copy of the Rey complex figure with immediate recall falling at 62nd centile and delayed recall falling at 82nd centile.

On cognitive processing speed, his score fell at 50th centile (digit symbol coding). On test of attention/executive function he scored normally on the trail-making test (part A = 91st centile, part B = 56th centile). Similarly on the Stroop task his score fell at the 75th centile. On a letter fluency task his score fell at the 16th centile, however category fluency task at the 82nd centile. His performance was entirely normal on both the Wisconsin card sorting task and the Iowa gambling task. He completed the Behavioural Assessment of the Dysexecutive Syndrome (BADS DEX) questionnaire scoring 29 and the independent rater score provided by his partner fell at 44. His partner also completed the neuropsychiatric inventory obtaining a total score of 14 with caregiver distress 11. His performance was entirely normal on the Key search task from the BADS, and on both components of the Hayling and Brixton tests. He scored within normal range on both indices of the Hospital Anxiety and Depression Scale (Anxiety = 5, Depression = 6).

### 4.3 Learning task

The learning task involved two options whose value varied independently, as in a two-armed bandit task. Participants were seated 70 cm in front of a touch-screen computer subtending approximately 70 degrees of visual arc, in a quiet dimly-lit room. They were instructed that they had to select one of two colours, red or blue. Each colour was associated with a probability of winning, but they would not be told the probabilities, and had to pay attention to the outcomes in order to know how good each colour was. Moreover the probabilities of each colour could change over time. It was explained that the values of the colours were independent, such that sometimes both red and blue might be winners, and at other times both might be bad. After selecting one of the colours, participants had to place a bet indicating how much they would win or lose. The independent probabilities of the two options ensures that it is not always optimal to bet high (Schurger & Sher, 2008), and participants were informed that they might sometimes have to bet low, for example if they were expecting to lose. Finally, after the outcome of the bet, they were required to rate their surprise at the outcome on a visual analogue scale. The scale was marked with phrases indicating degrees of surprise, and was not shown on the first 10 trials.

### 4.4 Materials

At the top of the screen, the “bank” displayed the amount of money accumulated so far in the block. Two coloured squares were shown at the top left and top right of the screen (Fig. 1A). After a touch was detected within the boundary of a square, that square was highlighted with a yellow outline with an audible click. After 500 ms, a row of five grey discs with monetary values in white text were displayed, with values “£1”, “£2”, “£5”, “£10” and “£20”. Participants were required to touch one disc, resulting in a yellow outline around the selected disc, and an audible click. After 500 ms, an outcome was displayed below these discs, either “Win £x” or “Lose £x”, with the selected stake inserted, and accompanied by either a high-pitched or low-pitched sound for wins and losses respectively. After a further 500 ms, a “surprise rating” linear analogue scale was displayed below the outcome, with the question “How much did you expect this?”, with five zones on the scale marked “Very surprised”, “A bit surprised”, “Unsurprised”, “Expected” and “I knew it!”. After touching a point on this scale, a vertical tick appeared on the scale, with an audible click. After 500 ms, the next trial began. Surprise ratings were only required for trials 10 and onwards in the experiment, so that participants could build an expectation of the outcomes before being asked to report surprise.

### 4.5 Design

The experiment constituted 160 trials, broken into 3 blocks, and the bank was initialised to £0 at the start of each block. The left/right location of the red and blue squares was randomised. The outcome on a given trial was chosen pseudorandomly using the probability currently associated with the chosen colour. These probabilities for each colour varied over time as follows. Each colour could win 70% of the time, or 30% of the time. These probabilities switched every 8 or 16 trials (mean 12 trials) (Fig. 1B). The probabilities of winning for each colour changed in a predetermined sequence that was designed to include each possible “change” type once. This meant that, on half of the changes, the probability associated with a colour stayed the same, or changed (i.e. from 70 to 30% or 30–70%). The transitions were balanced so that over the first 128 trials, participants experienced each of the four probability combinations (e.g. “blue = 70, red = 30”) for an equal amount of time, and also each of the 16 possible transitions an equal number of times.

### 4.6 Analysis

Choices of colour on each trial were fitted to a logistic model in which the relative values of the options were updated according to the outcomes on previous trials. A standard Rescorla-Wagner value-learning rule was used, in which the value of the chosen item was updated according to whether it won or lost:

$$Q_{t+1}^{c}\leftarrow Q_{t}^{c}+\alpha \left(R_{t}-Q_{t}^{c}\right)$$

where $Q_{t}^{c}$ is the value of the chosen item on trial *t*, and the reward $R_{t}$ is 0 or 1 to indicate a win, irrespective of the bet. The unchosen item's value is not updated,

$$Q_{t+1}^{u}\leftarrow Q_{t}^{u}$$

where $Q_{t}^{u}$ the value of the unchosen item. Choice proceeds according to a softmax rule

$$choose_{t}^{c}\;∼\;\beta \cdot \left(Q_{t}^{c}-Q_{t}^{u}\right)$$

with a logistic choice function. Equations: (1) reward prediction updating for the chosen item, (2) unchosen item's value is unchanged, (3) softmax rule to select an option. There are two free parameters, the learning rate α and the inverse temperature β. Models were fitted using maximum likelihood, fitted using a Gibbs sampler (JAGS).

To assess the effects of modelled value on betting and surprise ratings, we then used mixed effects linear models, fitted in MATLAB using fitlme (). Variables used as predictors were z-scored within subjects and a random intercept was always included. These models were used to examine how people chose to bet, and rated their surprise, based on their previous experiences. The model thus factors out the fact that different people may have different means and scaling of their bets, surprise ratings, and subjective values, and focuses only on relationships of within-subject trial-to-trial variation in these values. For the linear models, we used an ordinal scale from 1 to 5 for the bets. Since the spacing of the five bet options were approximately logarithmically spaced, this corresponds approximately to the log-bet. Fixed effects are quantified as t-statistics, yielding a 2-tailed p-value for each factor of interest.

To visualise these effects, choices and bets were plotted as a function of the modelled values on each trial inferred from the Rescorla-Wagner learner. Since each person had a different range of modelled values, the values were binned according to their quantile for each subject. Choices, bets and surprise ratings were averaged for each subject, within each bin. Then the mean and standard error across subjects was plotted for each bin. Bins were calculated using a sliding window of 25 percentiles. The x-coordinate for plotting each bin is the mean of the bin centres for each subject (Fig. 4A,C,D). This method corresponds roughly to the mixed models’ inclusion of a random intercept. However note that this is only to visualise the results, and all statistics were performed using the mixed models above. No part of the study procedures or analyses was preregistered in an institutional registry prior to the research being conducted.

### 4.7 Prosaccade, antisaccade and incentivised oculomotor capture tasks

The patient performed two saccadic tasks: pro-saccades and anti-saccades. For the prosaccade task, participants had to shift gaze to a visual target, as it moved from the centre of the screen either to the left or right side, amplitude 11.4°. Dim placeholders were always visible at the target locations. The antisaccade task was identical, except that participants were instructed to look to the opposite side to where the target appeared. Performance was compared to the data from 21 controls and the 19 medial prefrontal patients reported in Manohar and Husain, 2016, which included 3 patients who did not complete the learning task. Visual targets and were 4° in diameter and shown in 50% grey on a CRT monitor at 100 Hz, 60 cm from the eye. Participants sat in a chin and forehead rest, while eye position was recorded using a tower-mounted Eyelink 1000. 9-point calibration was used. Participants performed 96 trials of each task, split into two blocks.

Saccadic reaction time, amplitude and peak velocity were measured (Table 1). In the antisaccade task, when a bright visual stimulus appeared either on the left or right, participants were required to shift gaze in the opposite direction, to a dim placeholder. The error rate (proportion of saccades made towards the stimulus) and cost (difference in RT between antisaccades and prosaccades) were measured.

### 4.8 Working memory task

The patient and healthy controls performed a computerised visuospatial working memory task, a touch-screen analogue of the Corsi blocks task. Participants viewed a sequence of 1–6 dots, each dot lasting 500 ms followed by a 500 ms blank screen. After the sequence, there was a delay of 1 s, then participants were instructed to recall the sequence of locations. They had to touch the computer screen to indicate the remembered location of each dot. During recall, locations that had been touched were marked with a dot that remained visible.

Memory performance was quantified as the mean distance of each response from the corresponding presented dot, indicating the overall memory error. Statistics are reported on the logarithm of the mean error distance. The patient's performance was not significantly different to controls (Table 1). Unilateral lesion patients did not perform this working memory task but digit span showed no significant differences from controls (reported previously in Manohar & Husain, 2016).

## Credit author statement

SGM–Conceptualisation, Investigation, Writing, PL –Writing, DD–Investigation, SJF–Conceptualisation, Methodology, Writing, TTJC–Investigation, DSJ–Resources, IB–Invstigation, Resources, MH–Conceptualisation, Writing, Resources.

## Open practices

The study in this article earned Open Materials Open Data badges for transparent practices. Materials and data for the study are available at https://osf.io/4kfqz/.
