# Supplementary material for: Reduced decision bias and more rational decision making following ventromedial prefrontal cortex damage
Source: Cortex. 2021 May;138:24–37. doi: 10.1016/j.cortex.2021.01.015 (PMC8064028; doi:10.1016/j.cortex.2021.01.015)
Supplement: Multimedia component 1 [file mmc1.docx]

# Supplementary Methods

To fit the Rescorla-Wagner learning model, we used a Gibbs sampler (Just Another Gibbs Sampler, JAGS, Plummer, 2003) to fit each subject’s trials independently. The prior on α was a beta distribution with shape parameters *a*=5, *b*=10. The prior on β was a gamma distribution with shape parameters 2 and 0.2. The *Q*-values of both options were initialised to 0.5 at the start of the trial. Decisions between the two options were based on $Q_{1}-Q_{2}$, which ranged between -1 and +1. Outcomes were represented by a reward of 1 or 0, and learning only took place for the chosen option. The maximum a posteriori (MAP) estimates for the parameters and for the *Q*-values of each option at each trial were extracted. The ‘chosen value’ on each trial was the MAP estimate of $Q^{c}$ for the option chosen by the participant on that trial, and the unchosen value was the MAP of the other option $Q^{u}$.

# Supplementary analysis

**Bets increased after wins, when previous bets were low**

We modelled bets with the following:

$$bet_{t} \sim\left( 1+ Q_{t}^{c}+bet_{t-1}*win_{t-1} \right)*patient+\left( 1 \right| subject)$$

This model is similar to the one presented at L322, but with the addition of the previous-win term. Here, the $win_{t-1}$ term quantifies whether people bet more after a loss (corrected for the effect of the previous bet), and the $win_{t-1}*bet_{t-1}$ interaction quantifies whether the bias of the previous bet is weakened by a loss. We find that not only did people bet higher when they had previously bet high (as in Fig.4B), but also after winning on the previous trial (by 0.14 bet units, t=4.25, p<0.001). This boost from winning was smaller if people had already previously bet highly (previous bet * previous win interaction, 0.08 bet units less, t=-3.18, p=0.001). Importantly, the previous-win effect was not different between controls and unilateral patients (previous win *patient interaction, t=0.60, p=0.54), nor was the interaction with bets (three-way previous win * previous bet * patient interaction, t=-0.20, p=0.84). The previously observed effect that unilateral patients were biased less by the previous bet remained highly significant (previous bet * patient interaction, t=-4.69, p<0.001).

**
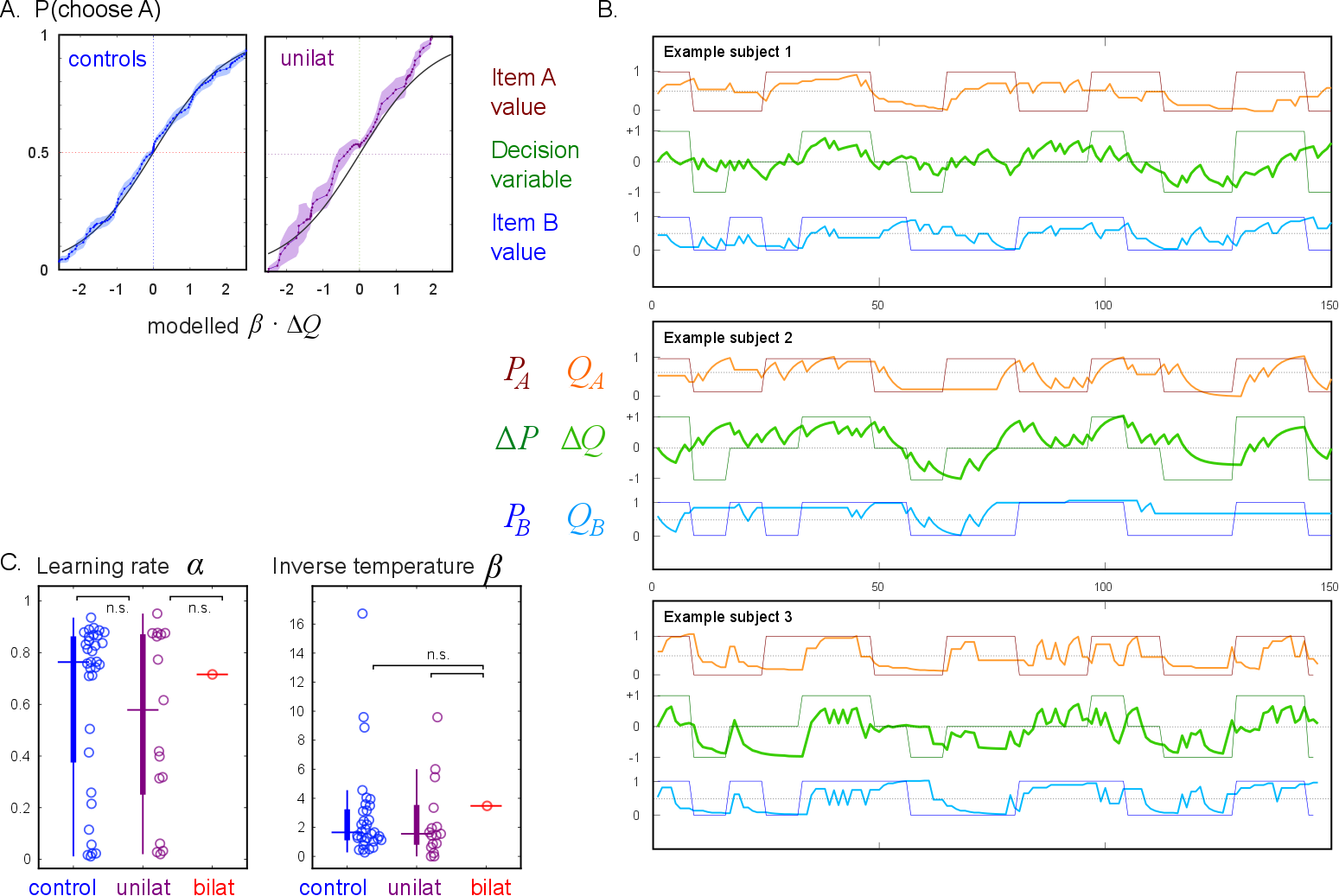
 Figure S1: Simple learning model fitted to data**

**A**: Choices of the ‘A’ option, as a function of modelled value. The solid black curves indicate the theoretical logistic curve, and points indicate the mean and standard error of proportion of choices across participants, taken in a sliding window of 20% quantiles over the modelled value.

**B**: Example traces of modelled value as a function of trial number for three participants with different parameters. On the y-axis is plotted: value of option A (orange), value of option B (blue), relative value A minus B (green). The thin lines indicate the actual probability assigned to the options in the task.

**C**: Fitted values of parameters, for each group, showing no differences in the learning or decision-making parameters. There were no significant differences in learning rates between patients and controls (mean $\alpha$=0.50 ± s.d. 0.37 in patients, *vs*. 0.68±0.31 in controls, two-sample *t*(47)=1.87, p=0.067; $\beta$=2.36±2.37 in patients *vs*. 1.98±1.41 in controls, t(47)=0.71, p=0.48), or between controls and the bilateral patient (patient $\alpha=0.88$, Z=1.03, p=0.30; $\beta=2.56$, Z=0.086, p=0.93), or between unilateral patients and the bilateral patient (*α*: Z=0.70, p=0.48; *β*: Z=0.59 p=0.56).

| Area | Proportion of template covered by lesion % | Proportion of lesion that overlaps the template % |
| --- | --- | --- |
| 11m L | 96 | 19 |
| 11m R | 98 | 20 |
| 14c L | 20 | 4 |
| 14c R | 14 | 3 |
| 14m L | 40 | 13 |
| 14m R | 34 | 11 |
| 14r L | 81 | 11 |
| 14r R | 67 | 10 |
| 14rr L | 96 | 13 |
| 14rr R | 95 | 10 |
| 24 L | 0.2 | 0 |
| 24 R | 0 | 0 |
| 25 L | 0 | 0 |
| 25 R | 0 | 0 |
| 32 L | 2 | 0.2 |
| 32 R | 2 | 0.3 |

**Table S1**: Projections of MJ’s lesion onto McGill-McConnell vmPFC atlas. Proportions indicate the inner product of the binary lesion with the probabilistic template, normalised either by the integral of the template probability (column 1) or by the lesion volume (column 2).

|  | **HC** (N=21) | | **Unilat** (N=19) | | **MJ (Bilat)** | *Z*-score MJ vs control | *p* |
| --- | --- | --- | --- | --- | --- | --- | --- |
|  | mean | s.d. | mean | s.d. |  |  |  |
| Prosaccade Accuracy (%) | 95.5 | 5.9 | 94.5 | 8.3 | 97.0 | 0.248 | 0.80 |
| Antisaccade Accuracy (%) | 71.6 | 14.5 | 75.9 | 14 | 70.0 | 0.107 | 0.92 |
| Prosaccade RT (ms) | 225 | 37.7 | 224 | 35.1 | 281 | 1.49 | 0.14 |
| Antisaccade RT (ms) | 344 | 71.1 | 311 | 63.4 | 411 | 0.95 | 0.34 |
| WM error (deg) | 4.75 | 0.41 |  |  | 4.84 | 0.20 | 0.84 |

**Table S2**: Performance of the bilateral patient on simple prosaccades, antisaccades and a visuospatial working memory task. There were no significant differences from healthy controls on these tasks. Note the unilateral group data on this task (reported in Manohar et al 2016) includes 3 patients who did not perform the learning task (total N=19), and a different group of controls. WM: Visuospatial working memory task.
